# Circulating plasma microRNAs as potential markers to identify *EGFR* mutation status and to monitor epidermal growth factor receptor-tyrosine kinase inhibitor treatment in patients with advanced non-small cell lung cancer

**DOI:** 10.18632/oncotarget.17416

**Published:** 2017-04-25

**Authors:** Lili Qu, Liangliang Li, Xiaofei Zheng, Hanjiang Fu, Chuanhao Tang, Haifeng Qin, Xiaoyan Li, Hong Wang, Jianjie Li, Weixia Wang, Shaoxing Yang, Lin Wang, Guanhua Zhao, Panpan Lv, Yangyang Lei, Min Zhang, Hongjun Gao, Santai Song, Xiaoqing Liu

**Affiliations:** ^1^ Department of Lung Cancer, Affiliated Hospital of the Academy of Military Medical Science, Beijing, China; ^2^ Institute of Radiation Medicine, Academy of Military Medical Science, Beijing, China; ^3^ Department of Oncology, Peking University International Hospital, Beijing, China; ^4^ Department of Medical Oncology, Beijing Cancer Hospital, Beijing, China; ^5^ Department of Breast Cancer, Affiliated Hospital of the Academy of Military Medical Science, Beijing, China

**Keywords:** circulating microRNA, *EGFR* mutation status, epidermal growth factor receptor-tyrosine kinase inhibitor, non-small cell lung cancer, tumor marker

## Abstract

We aimed to identify a panel of circulating plasma microRNAs that can predict *EGFR* mutation status and monitor epidermal growth factor receptor-tyrosine kinase inhibitor treatment in patients with non-small cell lung cancer. Microarrays were performed for the preliminary screening of dysregulated microRNAs in 9 *EGFR* mutation-positive patients *versus* healthy controls. MiR-107 was upregulated and miR-195 was downregulated in the exon 19 deletion *versus* wild-type group. The areas under the receiver operating characteristic curves for miR-107, miR-195, and a panel of these 2 microRNAs were 0.72, 0.75, and 0.74, with sensitivities and specificities of 64.7% and 76.6%, 71.8% and 69.1%, and 71.7% and 78.9%, respectively. MiR-122 was significantly upregulated in the p.L858R *versus* wild-type group. An area under the receiver operative characteristic curve of 0.75 suggests that miR-122 might be a specific biomarker for patients with the p.L858R mutation. In addition, dynamic changes in these 3 microRNAs were also found to correlate with responses to epidermal growth factor receptor-tyrosine kinase inhibitor treatment, indicating that circulating plasma microRNAs may represent potential biomarkers for monitoring epidermal growth factor receptor-tyrosine kinase inhibitor treatment. This study demonstrates the prospective application of circulating plasma microRNAs as potential non-invasive, convenient biomarkers for patients with *EGFR*-sensitive mutations.

## INTRODUCTION

As one of the most frequently diagnosed cancers, lung cancer continues to represent the first leading cause of cancer-related mortality worldwide [1, 2]. Non-small cell lung cancer (NSCLC) accounts for approximately 80.0–85.0% of all lung cancers and remains a significant public health problem in China [1]. The majority of NSCLC patients are diagnosed at advanced stages with poor prognoses, especially in those treated with traditional chemotherapy regimens [3].

To date, the *EGFR* gene remains the most important oncogenic driver of NSCLC and treatment-naïve patients with advanced NSCLC harboring specific *EGFR*-sensitive mutations (principally *EGFR* exon 19 deletions and *EGFR* exon 21 p.L858R point mutations that account for approximately 45.0% and 40.0% of patients, respectively) are recommended to receive first-line epidermal growth factor receptor-tyrosine kinase inhibitors (EGFR-TKIs) as per the National Comprehensive Cancer Network guidelines [4–6]. The efficacy of EGFR-TKIs has been proven in several large-scale randomized clinical trials, especially for Asian, female, non-smoking individuals and adenocarcinoma patients [7]. Specifically, the *EGFR* exon 19 deletion was reported to be associated with a longer progression-free survival (PFS) compared to the *EGFR* p.L858R point mutation [8]. Therefore, it is crucial to identify the genotype of the tumor after the histopathological classification is determined to predict the sensitivity or resistance to an increasing number of EGFR-TKIs.

In a clinical setting, obtaining adequate tumor specimens for pathological evaluation and specific molecular analyses are decisive prerequisites for establishing an optimal, individualized treatment regimen for the patient. Tumor biopsy specimens can sometimes be difficult to obtain from certain patients. Tumor pathology and genotyping are often determined prior to commencing first-line EGFR-TKI treatment. However, re-biopsy during or after the time of progressive disease (PD) on EGFR-TKI treatment to continuously monitor *EGFR*-sensitive mutations and to analyze the mechanism(s) of resistance to EGFR-TKI treatment remains a significant challenge and appears to be unrealistic. Hence, as a non-invasive test, liquid biopsy has warranted wide attention in recent years with its unique advantage of simultaneously capturing multiple sites of tumor growth and testing *EGFR* mutation status, which could assist oncological clinicians in the timely adjustment of therapeutic strategies for NSCLC patients [9, 10]. Therefore, it is imperative to explore non-invasive, convenient, and economical tumor markers to predict *EGFR* mutation status and to monitor EGFR-TKI treatment in NSCLC patients.

MicroRNAs comprise a large family of small (approximately 21–25 nucleotides in length) endogenous, non-coding RNAs that negatively regulate gene expression at post-transcriptional level via inhibition of target messenger RNAs by pairing with the complementary sequences in the 3′ untranslated region [11, 12]. MicroRNAs exert a wide range of biological functions, including early tumorigenesis and tumor progression. It has been reported that circulating microRNAs are packaged into microparticles or are associated with RNA binding proteins and lipoprotein complexes, making them ideal candidates for tumor biomarkers owing to their high stability in body fluids [13, 14]. Accumulating evidence has proven that circulating microRNA signatures in human plasma or serum may serve as disease fingerprints and novel molecular markers for NSCLC [15–17]. However, associations between circulating microRNAs in plasma and *EGFR* mutation status and their application for monitoring EGFR-TKI treatment and disease progression have not been systematically studied. Therefore, we aimed to identify a panel of circulating plasma microRNAs that can distinguish between NSCLC patients with *EGFR*-sensitive mutations and *EGFR* wild-type patients and to explore the potential of this microRNA panel to monitor tumor responses to EGFR-TKI treatment.

## RESULTS

### Patient characteristics

Between December 2014 and April 2016, we recruited 153 patients with pathologically confirmed NSCLC and 41 healthy controls, with a median age of 56.1 (range, 45–78) years and a male to female ratio of 0.71. The clinical characteristics of the patients are summarized in Table 1.

**Table 1:** Clinical characteristics of NSCLC patients (*n* = 153)

Characteristic	NSCLC patients				*p*-value^b^
	*EGFR*^19DEL^ (*n* = 64)	*EGFR*^p.L858R^ (*n* = 36)	*EGFR*^MUT(+)a^ (*n* = 100)	*EGFR*^WT^ (*n* = 53)	
Age (years), mean (range)	56.3 (37–76)	61.1 (34–80)	57.9 (34–80)	59.0 (34–84)	0.537
Sex, *n* (%)					
M	29 (45.3)	17 (47.2)	46 (46.0)	26 (49.1)	
F	35 (54.7)	19 (52.8)	54 (54.0)	27 (50.9)	0.424
ECOG PS, *n* (%)					
0	0 (0.0)	0 (0.0)	0 (0.0)	0 (0.0)	
1	64 (100.0)	36 (100.0)	100 (100.0)	53 (100.0)	
2	0 (0.0)	0 (0.0)	0 (0.0)	0 (0.0)	N/A
Complications, *n* (%)					
Y	17 (26.6)	9 (25.0)	26 (26.0)	18 (34.0)	
N	47 (73.4)	27 (75.0)	74 (74.0)	35 (66.0)	0.198
Smoker, *n* (%)					
Y	22 (34.4)	13 (36.1)	35 (35.0)	28 (52.8)	
N	42 (65.6)	23 (63.9)	65 (65.0)	25 (47.2)	0.025*
FH of cancer, *n* (%)					
Y	8 (12.5)	5 (13.9)	13 (13.0)	10 (18.9)	
N	56 (87.5)	31 (86.1)	87 (87.0)	43 (81.1)	0.231
cStage, *n* (%)					
IIIA–B	7 (10.9)	3 (8.3)	10 (10.0)	9 (17.0)	
IV	57 (89.1)	33 (91.7)	90 (90.0)	44 (83.0)	0.161
Histology, *n* (%)					
ADC	59 (92.2)	34 (94.4)	93 (93.0)	51 (96.2)	
ADC+SCC	5 (7.8)	2 (5.6)	7 (7.0)	2 (3.8)	0.340

* *p* < 0.05

^a^ *EGFR*^19DEL^ and *EGFR*^p.L858R^ patients combined

^b^EGFR^MUT(+)^ versus EGFR^WT^

Abbreviations: 19DEL, exon 19 deletion; ADC, adenocarcinoma; cStage, clinical stage; ECOG, Eastern Cooperative Oncology Group; F, female; FH, family history; M, male; MUT(+), mutation-positive; N, no; N/A, not applicable; PS, performance status; SCC, squamous cell carcinoma; WT, wild-type; Y, yes

Briefly, plasma samples were collected from 64 *EGFR* exon 19 deletion, 36 *EGFR* p.L858R mutation, and 53 *EGFR* wild-type patients in the Department of Lung Cancer and 41 healthy controls from the Physical Examination Centre at our institution. With the exception of smoking status, no significant differences in clinical characteristics were observed between the NSCLC patients and healthy controls or between subgroups of the NSCLC patients stratified according to *EGFR* mutation status. Groups containing patients with *EGFR-*sensitive mutations (*EGFR* exon 19 deletions or *EGFR* p.L858R point mutations) had fewer smokers than the group containing *EGFR* wild-type patients (*p* < 0.05). Blood samples of 36 of the 64 *EGFR* exon 19 deletion patients were dynamically collected at hospital visits at 1, 3, and 5 months during EGFR-TKI treatment. In addition, blood samples of 12 patients were also collected at the time of PD on EGFR-TKI treatment (Table 2).

**Table 2:** Clinical characteristics of *EGFR* exon 19 deletion patients with NSCLC who had dynamic plasma collected (*n* = 36)

Characteristic	Patients (*n* = 36)
Age (years), mean (range)	51.3 (41–72)
Sex, *n* (%)	
M	17 (47.2)
F	19 (52.8)
ECOG PS, *n* (%)	
0	0 (0.0)
1	36 (100.0)
2	0 (0.0)
Complications, *n* (%)	
Y	9 (25.0)
N	27 (75.0)
Smoker, *n* (%)	
Y	7 (19.4)
N	29 (80.6)
FH of cancer, *n* (%)	
Y	3 (8.3)
N	33 (91.7)
cStage, *n* (%)	
IIIA–B	2 (5.5)
IV	34 (94.5)
Histology, *n* (%)	
ADC	35 (97.2)
ADC+SCC	1 (2.8)
EGFR-TKI treatment, *n* (%)	
Gefitinib	19 (52.8)
Erlotinib	1 (2.8)
Icotinib	16 (44.4)
Line of EGFR-TKI treatment, *n* (%)	
First	28 (77.8)
Second	8 (22.2)
Best response to EGFR-TKI treatment, *n* (%)	
CR	1 (2.8)
PR	23 (63.9)
SD	11 (30.5)
PD	1 (2.8)
Sample collected during EGFR-TKI treatment, *n* (%)	
Y	36 (100.0)
N	0 (0.0)
Sample collected at the time of PD on EGFR-TKI treatment, *n* (%)	
Y	12 (33.3)
N	24 (66.7)

Abbreviations: ADC, adenocarcinoma; CR, complete response; cStage, clinical stage; ECOG, Eastern Cooperative Oncology Group; F, female; FH, family history; M, male; N, no; PS, performance status; SCC, squamous cell carcinoma; Y, yes

### Microarray analysis of candidate plasma microRNAs for *EGFR*-sensitive mutations

Plasma samples of 3 *EGFR* exon 19 deletion, 3 *EGFR* p.L858R mutation, and 3 *EGFR* wild-type patients and 4 healthy controls were selected for Agilent microRNA microarray analysis to detect differences in the expression levels of circulating plasma microRNAs (*n* = 2,568) between the above cohorts (Gene Expression Omnibus accession number: GSE93300).

**Figure 1:**
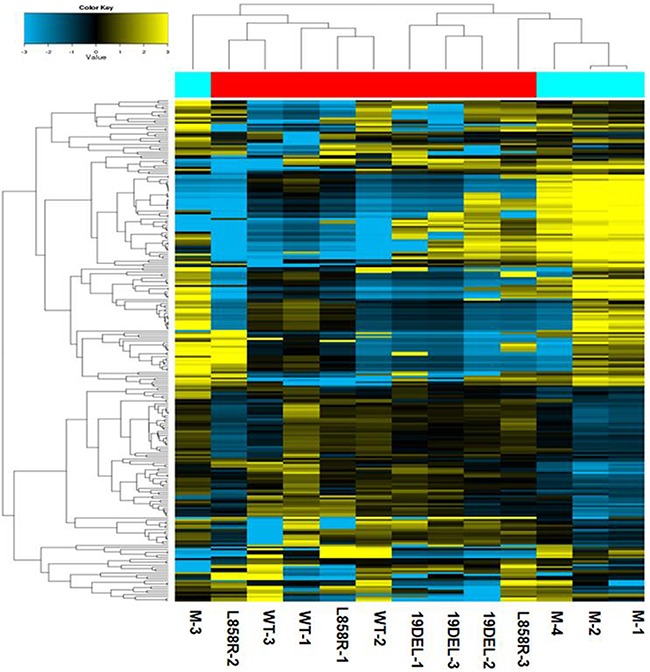
Hierarchical clustering analysis of circulating microRNA expression in plasma from patients with NSCLC *versus* healthy controls M-1, M-2, M-3, and M-4 represent the healthy controls.

Compared to healthy controls, 19 microRNAs were upregulated and 57 microRNAs were downregulated in the plasma of all 9 NSCLC patients (fold-change >2.0, *p* < 0.05) (Figure 1). Further analysis of the 76 dysregulated microRNAs was performed to select candidate microRNAs between NSCLC patients with different *EGFR*-sensitive mutations. Compared to the *EGFR* wild-type group, 14 microRNAs were up-regulated and 7 microRNAs were downregulated in *EGFR* exon 19 deletion group, and 2 microRNAs were upregulated and 3 microRNAs were downregulated in the *EGFR* p.L858R mutation group (fold-change >2.0, *p* < 0.05; Table 3 and Table 4). It is noteworthy that relative to the *EGFR* wild-type group, the *EGFR* exon 19 deletion group contained more differently expressed microRNAs than the *EGFR* p.L858R mutation group. Indeed, it has been reported that *EGFR* exon 19 deletion patients are associated with a longer PFS compared to *EGFR* p.L858R mutation patients [8]. Thus, we primarily focused on *EGFR* exon 19 deletion patients in our subsequent analyses.

**Table 3:** Microarray analysis of the differential expression of circulating plasma microRNAs in *EGFR* exon 19 deletion *versus EGFR* wild-type NSCLC patients

MicroRNA	Fold-change	Regulation	*p*-value
miR-25-3p	36.0	Up	0.002*
miR-15b-5p	14.9	Up	0.005*
let-7b-5p	10.6	Up	0.020*
miR-21-5p	16.1	Up	0.046*
miR-4306	42.6	Up	0.001*
miR30d-5p	34.5	Up	0.006*
let-7i-5p	27.5	Up	0.003*
miR-19a-3p	27.7	Up	0.002*
miR-17-5p	12.3	Up	0.010*
miR-20a-5p	31.1	Up	0.003*
miR-3195	8.5	Up	0.038*
miR-24-3p	32.9	Up	0.008*
miR-15a-5p	24.6	Up	0.004*
miR-107	30.2	Up	0.002*
miR-6751-3p	32.9	Down	0.046*
miR-6779-3p	14.3	Down	0.022*
miR-6858-5p	30.0	Down	0.036*
miR-7106-5p	21.3	Down	0.033*
miR-6797-3p	17.4	Down	0.030*
miR-483-3p	30.2	Down	0.039*
miR-619-5p	10.9	Down	0.021*

* *p* < 0.05

**Table 4:** Microarray analysis of the differential expression of circulating plasma microRNAs in *EGFR* p.L858R mutation *versus EGFR* wild-type NSCLC patients

MicroRNA	Fold-change	Regulation	*p*-value
miR-1229	21.9	Up	0.017*
miR-3141	9.3	Up	0.027*
miR-4281	2.8	Down	0.010*
miR-4516	3.9	Down	0.008*
miR-3663-3p	3.0	Down	0.035*

* *p* < 0.05

### Quantitative real-time reverse-transcription polymerase chain reaction (qRT-PCR) validation of candidate stable circulating plasma microRNAs

In total, 20 NSCLC patients (*EGFR* exon 19 deletion [*n* = 12] and *EGFR* wild-type [*n* = 8]) and 8 healthy controls were selected for further screening of candidate microRNAs correlating with *EGFR* exon 19 deletion mutation status. Ten microRNAs were screened out from the above microarray data set (miR-107, miR-19a-3p, miR-20a-5p, miR-21-5p, miR-24-3p, miR-25-3p, and miR-30d-5p) and published literature (miR122, miR-125a-5p, and miR-195) [15–17] using qRT-PCR in a pre-experiment. The 5S ribosomal RNA (5SrRNA) was used as an endogenous control to normalize the raw quantification cycle (Ct) values. Subtraction of the Ct value of the target microRNA from 5SrRNA (ΔCt) was performed and converted to 2^−ΔCt^ to assess alterations in microRNA expression levels between the cohorts. Fold-changes were calculated using the 2^−Δ(ΔCt)^ method, where Δ(ΔCt) = (Ct_miR_ – Ct_5SrRNA_)_EGFR-MUT(+)_ – (Ct_miR_ – Ct_5SrRNA_)_EGFR-WT_. Table 5 displays the relative expression levels of the 10 plasma microRNAs in the *EGFR* exon 19 deletion *versus EGFR* wild-type group. We found that miR-19a-3p, miR-20a-5p, miR-21-5p, miR-24-3p, miR-25-3p, and miR-30d-5p were not significantly differentially expressed in the *EGFR* exon 19 deletion *versus EGFR* wild-type group. Therefore, 4 microRNAs (miR-107, miR-122, miR-125a-5p, and miR-195) were selected for further investigation.

**Table 5:** Pre-experimental analysis of the differential expression of 10 selected circulating plasma microRNAs in *EGFR* exon 19 deletion *versus EGFR* wild-type NSCLC patients

MicroRNA	Fold-change^a^	Regulation	*p*-value
miR-19a-3p	4.1	N/A	0.070
miR-20a-5p	5.7	N/A	0.052
miR-21-5p	4.8	N/A	0.107
miR-24-3p	4.0	N/A	0.119
miR-25-3p	5.7	N/A	0.052
miR-30d-5p	4.1	N/A	0.098
miR-107	14.5	Up	0.002*
miR-122	6.3	Up	0.005*
miR-125a-5p	6.6	Up	0.017*
miR-195	9.0	Down	0.002*

* *p* < 0.05

^a^Calculated using the equation 2^−Δ (ΔCt)^, where Δ(ΔCt) = (Ct_miR_ – Ct_5SrRNA_)_EGFR-MUT(+)_ – (Ct_miR_ – Ct_5SrRNA_)_EGFR-WT_

Abbreviations: N/A, not applicable.

### Dysregulated circulating plasma microRNAs for *EGFR*-sensitive mutations

**Figure 2:**
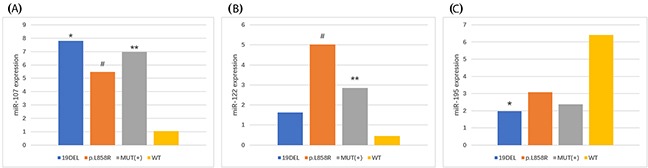
**(A)** MiR-107, **(B)** miR-122, and **(C)** miR-195 expression in NSCLC patients with different *EGFR*-sensitive mutations. *EGFR exon 19 deletion (19DEL) *vs*. *EGFR* wild-type (WT) patients (fold-change: miR-107, 7.44 and miR-195, 3.25; *p* < 0.05). ^#^*EGFR* p.L858R mutation *vs. EGFR*^WT^ patients (fold-change: miR-107, 5.22 and miR-122, 11.29; *p* < 0.05). ^*^EGFR mutation-positive (MUT[+]) *vs*. *EGFR*^WT^ patients (fold-change: miR-107, 6.64 and miR-122, 6.41; *p* < 0.05).

To further investigate the dysregulated circulating plasma microRNAs in NSCLC *EGFR* mutation-positive (*EGFR* exon 19 deletion or *EGFR* p.L858R mutation) patients, we performed qRT-PCR with 5SrRNA as the endogenous control for the *EGFR* exon 19 deletion, *EGFR* p.L858R mutation, and *EGFR* wild-type groups. MiR-125a-5p was screened out for exhibiting no statistically significant differences between the cohorts. MiR-107 was significantly upregulated in the *EGFR* exon 19 deletion, *EGFR* p.L858R mutation, and *EGFR* mutation-positive groups compared to the *EGFR* wild-type group (*p* < 0.05; Figure 2A and Table 6). Conversely, no statistically significant differences were observed between the *EGFR* exon 19 deletion and *EGFR* p.L858R mutation groups. MiR-122 was significantly upregulated in the *EGFR* p.L858R mutation group compared to the *EGFR* wild-type or *EGFR* mutation-positive groups (*p* < 0.05). Conversely, no statistically significant differences were observed between the *EGFR* exon 19 deletion and *EGFR* p.L858R mutation or *EGFR* wild-type groups (*p* > 0.05; Figure 2B and Table 6). MiR-195 was significantly downregulated in the *EGFR* exon 19 deletion group compared to the *EGFR* wild-type group (*p* < 0.05), although no statistically significant differences were observed between the other groups (*p* > 0.05; Figure 2C and Table 6).

**Table 6:** Relative expression of miR-107, miR-122, and miR-195 in *EGFR* exon 19 deletion (*EGFR*^19DEL^), *EGFR* p.L858R mutation (*EGFR*^p.L858R^), and *EGFR* wild-type (*EGFR*^WT^) patients with NSCLC (*n* = 153)

microRNA	Normalized expression (mean ± SEM)^a^				*p*-value			
	*EGFR*^19DEL^(*n* = 64)	*EGFR*^p.L858R^ (*n* = 36)	*EGFR*^MUT(+b^ (*n* = 100)	*EGFR*^WT^(*n* = 53)	19DEL *vs*. WT	p.L858R *vs*. WT	MUT(+)^b^ *vs*. WT	19DEL *vs*. p.L858R
miR-107	7.80 ± 2.88	5.47 ± 2.05	6.96 ± 1.98	1.05 ± 0.41	0.024*	0.041*	0.033*	0.513
miR-122	1.62 ± 1.12	5.02 ± 2.28	2.85 ± 1.10	0.45 ± 0.28	0.310	0.018*	0.036*	0.136
miR-195	1.98 ± 0.84	3.08 ± 0.97	2.37 ± 0.64	6.41 ± 2.01	0.033*	0.199	0.061	0.349

* *p* < 0.05

^a^Data expressed as the mean (2^−ΔCt^) ± SEM

^b^*EGFR*^19DEL^ and *EGFR*^p.L858R^ patients combined

Abbreviations: SEM, standard error of the mean

### Diagnostic analysis of circulating plasma microRNAs for *EGFR* mutation-positive NSCLC patients

**Figure 3:**
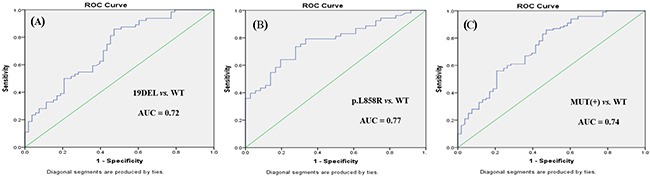
ROC curves for miR-107 in **(A)** *EGFR* exon 19 deletion (19DEL), **(B)** *EGFR* p.L858R mutation, and **(C)** *EGFR* mutation-positive (MUT[+]) patients with NSCLC *versus EGFR* wild-type (WT) patients.

**Figure 4:**
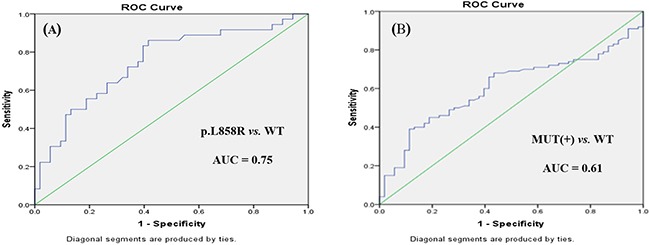
ROC curves for miR-122 in **(A)** *EGFR* p.L858R mutation and **(B)** *EGFR* mutation-positive (MUT[+]) patients with NSCLC *versus EGFR* wild-type (WT) patients.

**Figure 5:**
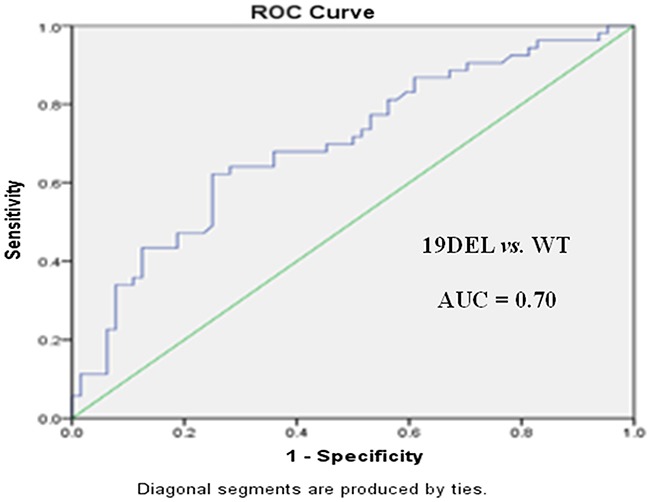
ROC curve for miR-195 in *EGFR* exon 19 deletion (19DEL) *versus EGFR* wild-type (WT) patients

Receiver operating characteristic (ROC) curve analysis was performed to evaluate the potential applications of circulating plasma miR-107, miR-122, and miR-195 as diagnostic markers for NSCLC patients with *EGFR*-sensitive mutations. MiR-107 exhibited a significant difference between *EGFR* mutation-positive and *EGFR* wild-type patients. In a comparison between the *EGFR* exon 19 deletion and *EGFR* wild-type groups, miR-107 was associated with an area under the ROC curve (AUC) value of 0.72 (95.0% confidence interval [CI]: 0.62–0.81), with a sensitivity of 64.7% and a specificity of 76.6% at a cutoff of 0.097 (Figure 3A). Meanwhile, in a comparison between the *EGFR* p.L858R mutation and *EGFR* wild-type groups, miR-107 was associated with an AUC value of 0.77 (95.0% CI: 0.68–0.87), with a sensitivity of 64.2% and a specificity of 80.6% at a cutoff of 0.153 (Figure 3B). With respect to miR-122, the AUC value was 0.75 (95.0% CI: 0.64–0.85), with a sensitivity of 73.6% and a specificity of 63.9% at a cutoff of 0.124, for the comparison between the *EGFR* p.L858R mutation and *EGFR* wild-type groups (Figure 4A). Nevertheless, despite the significant difference in miR-122 expression between the *EGFR* mutation-positive and *EGFR* wild-type groups, ROC curve analysis demonstrated that miR-122 was unable to distinguish between the two groups (AUC value: 0.61, 95.0% CI: 0.52–0.69; Figure 4B). However, miR-195 could distinguish between the *EGFR* exon 19 deletion and *EGFR* wild-type groups. The AUC value was 0.75 (95.0% CI: 0.60–0.79), with a sensitivity of 71.8% and a specificity of 69.1% at a cutoff of 0.876 (Figure 5).

**Figure 6:**
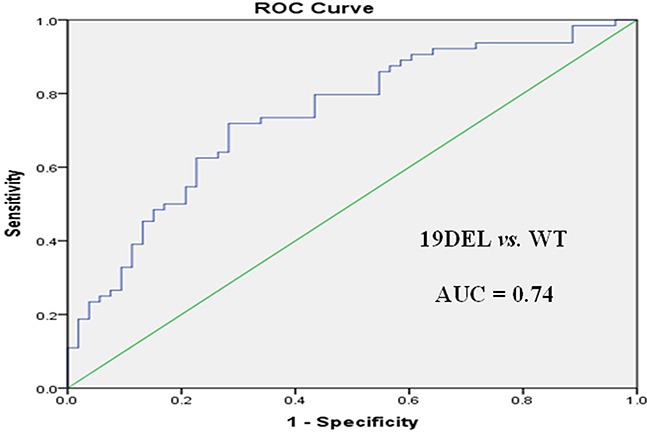
ROC curve for miR-107 and miR-195 in *EGFR* exon 19 deletion (19DEL) *versus EGFR* wild-type (WT) patients

**Figure 7:**
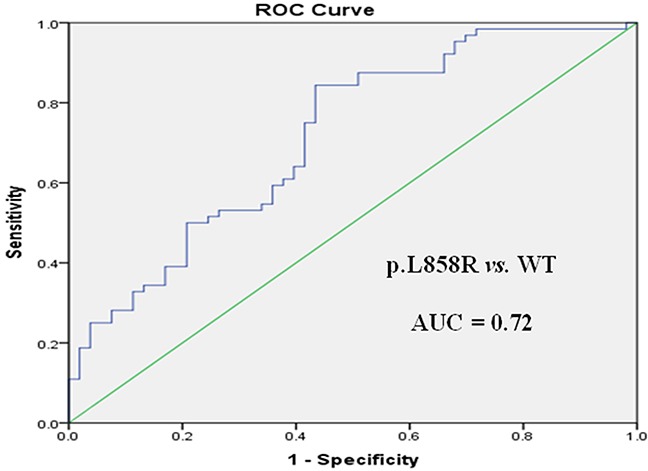
ROC curve for miR-107 and miR-122 in *EGFR* exon 19 deletion (19DEL) *versus EGFR* wild-type (WT) patients

To further evaluate the diagnostic potential of combinations of miR-107, miR-122, and miR-195, as panels, we performed logistic regression analysis combined with ROC curve analysis for these 3 microRNAs. A panel of miR-107 and miR-195 produced an AUC value of 0.74 (95.0% CI: 0.65–0.83), with a sensitivity of 71.7% and a specificity of 78.9% at a cutoff of -0.057, for the comparison between the *EGFR* exon 19 deletion and *EGFR* wild-type groups (Figure 6 and Table 7). This was higher than the AUC and sensitivity and specificity values of miR-107 or miR-195 alone as potential diagnostic markers. As for the comparison between the *EGFR* p.L858R mutation and *EGFR* wild-type groups, a panel of miR-107 and miR-122 produced an AUC value of 0.78 (95.0% CI: 0.69–0.88), with a sensitivity of 67.1% and a specificity of 82.5% at a cutoff of 0.321 (Figure 7 and Table 7). This was higher than the AUC value of miR-107 or miR-122 alone as markers to distinguish between these two groups.

**Table 7:** Measures of the diagnostic potential of the differentiation of NSCLC patients with *EGFR* exon 19 deletion (*EGFR*^19DEL^) or *EGFR* p.L858R mutations (*EGFR*^p.L858R^) *versus EGFR* wild-type (*EGFR*^WT^) patients

Variable	*EGFR*^19DEL^ *vs. EGFR*^WT^			*EGFR*^p.L858R^ *vs*. *EGFR*^WT^		
	miR-107	miR-195	miR-107 + miR-195	miR-107	miR-122	miR-107 + miR-122
AUC	0.72	0.75	0.74	0.77	0.75	0.78
Sensitivity	64.7%	71.8%	71.7%	64.2%	73.6%	67.1%
Specificity	76.6%	69.1%	78.9%	80.6%	63.9%	82.5%
Cutoff	0.097	0.876	−0.057	0.153	0.124	0.321
PPV	82.1%	79.0%	91.4%	80.0%	83.7%	81.8%
NPV	76.8%	80.3%	80.3%	89.8%	86.9%	88.3%

Abbreviations: PPV, positive predictive value; NPV, negative predictive value.

Meanwhile, we also estimated the positive and negative predictive values for distinguishing between NSCLC patients harboring different*EGFR*-sensitive mutations. In a comparison between the *EGFR* exon 19 deletion and *EGFR* wild-type groups, the positive and negative predictive values of a panel of miR-107 and miR-195 were 91.4% and 80.3%, respectively (Table 7). With respect to the *EGFR* p.L858R mutation *versus EGFR* wild-type group, the positive and negative predictive values of a panel of miR-107 and miR-122 were 81.8% and 88.3%, respectively. These results also indicate the potential diagnostic capability of the selected microRNAs.

### Univariate and multivariate analyses of clinicopathological variables associated with *EGFR*-sensitive mutations

In the univariate analysis, smoking status, miR-107, and miR-195 significantly correlated with *EGFR* mutation status (*EGFR* exon 19 deletion or p.L858R mutation *versus EGFR* wild-type) (Table 8). A multivariate analysis was subsequently performed to identify associations between miR-107, miR-195, and clinicopathological variables (sex, age, complications, and smoking status) and *EGFR*-sensitive mutations. Since we are in the Department of Medical Oncology, all of the enrolled patients were diagnosed with locally advanced or advanced stage disease. Meanwhile, the NSCLC patients we screened in this study were all newly diagnosed with an Eastern Cooperative Oncology Group performance status of 1. Consequently, clinical stage and Eastern Cooperative Oncology Group performance status were excluded from the multivariate analysis. The final results revealed that miR-107 and smoking status are important diagnostic predictors of *EGFR*-sensitive mutations. NSCLC patients with high levels of miR-107 expression and no prior history of smoking were more likely to harbor *EGFR* exon 19 deletions or *EGFR* p.L858R mutations.

**Table 8:** Univariate and multivariate analyses of clinicopathological variables associated with *EGFR*-sensitive mutations

Variable	Hazard ratio^a^	95.0% CI	*p*-value
**Univariate analysis**			
Age	−0.25	−4.05-3.13	0.811
Sex (male vs. female)	1.60	N/A	0.236
Complications (no *vs*. yes)	0.67	N/A	0.478
Smoker (no *vs*. yes)	10.58	N/A	0.002*
miR-107	2.15	0.49–11.34	0.033*
miR-122	1.58	−0.60–5.40	0.116
miR-195	−2.36	−7.41–-0.66	0.019*
**Multivariate analysis**			
Age	2.03	0.93–1.01	0.154
Sex (male *vs*. female)	1.08	0.63–4.62	0.299
Complications (no *vs*. yes)	2.55	0.86–4.53	0.110
Smoker (no *vs*. yes)	9.44	1.85–16.25	0.002*
miR-107	4.08	0.78–1.00	0.043*
miR-122	0.69	0.79–1.10	0.405
miR-195	2.81	0.99–1.07	0.094
**Further multivariate analysis**			
Smoker (no *vs*. yes)	9.90	1.55–6.67	0.002*
miR-107	5.56	0.78–0.98	0.018*

* *p* < 0.05

^a^Chi-square values for categorical variables and *t*-test values for continuous variables in the univariate analysis and Wald values for variables in the multivariate analysis

Abbreviations: N/A, not applicable

### Circulating plasma microRNAs as potential markers for monitoring EGFR-TKI treatment

**Figure 8:**
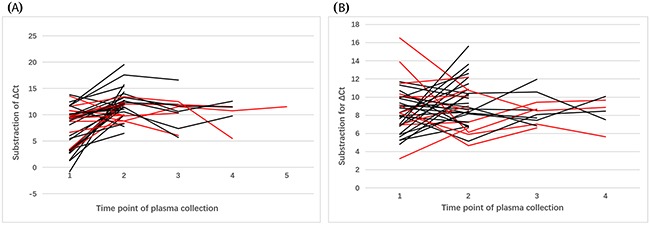
Dynamic changes in the expression of **(A)** miR-107 and **(B)** miR-195 in *EGFR* exon 19 deletion patients with NSCLC during EGFR-TKI treatment (*n* = 36). Patients with a complete or partial response are represented by the black lines and patients with stable disease are represented by the red lines.

**Figure 9:**
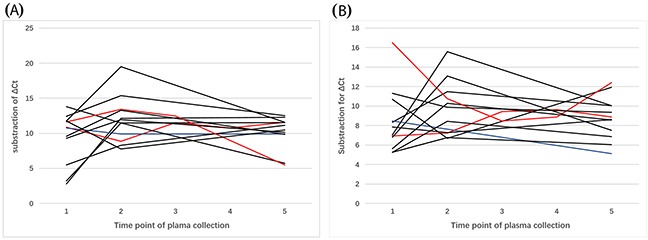
Dynamic changes in the expression of **(A)** miR-107 and **(B)** miR-195 in *EGFR* exon 19 deletion patients with NSCLC during and at the time of progressive disease on EGFR-TKI treatment (*n* = 12). Patients with a complete or partial response are represented by the black lines, patients with stable disease are represented by the red lines, and patients with progressive disease are represented by the blue lines.

As mentioned above, we collected plasma samples for a subset of patients harboring *EGFR*-sensitive mutations during EGFR-TKI treatment. The clinical characteristics and responses to EGFR-TKI treatment are summarized in Table 2. The median PFS was 8.0 (range, 1.0–31.0) months. To elucidate the fluctuations in circulating microRNAs with the course of EGFR-TKI treatment, subtraction of the ΔCt before EGFR-TKI treatment with that during or after the time of PD on EGFR-TKI treatment was analyzed. According to the Response Evaluation Criteria in Solid Tumors (version 1.1) [18], only one patient exhibited the poor response, PD. Consequently, the PD group was not analyzed in this comparison. As illustrated in Figure 8A, the ΔCt of miR-107 in patients with a complete or partial response (CR/PR) to EGFR-TKI treatment appeared to increase more sharply in comparison to that of patients with stable disease (SD) and this proved to be statistically significant (*p* < 0.05). Nevertheless, no statistically significant difference was detected between patients with a PFS of >8.0 months compared to patients with a PFS of ≤8.0 months for EGFR-TKI treatment (*p* > 0.05). Similarly, subtraction of the ΔCt for miR-195 revealed a significant difference between the CR/PR and SD groups (*p* < 0.05). This suggested that miR-195 expression in patients exhibiting a CR or PR to EGFR-TKI treatment could be significantly downregulated compared to patients exhibiting SD in response to EGFR-TKI treatment (Figure 8B). Meanwhile, for 12 patients, plasma was collected at the time of PD on EGFR-TKI treatment. Figure 9A and Figure 9B indicate that no matter the response of the tumor (CR, PR, SD, or PD), miR-107 and miR-195 expression levels at the time of PD exhibit a tendency to return to the levels before EGFR-TKI treatment in the majority of patients.

## DISCUSSION

“Precision medicine” warrants further attention in the individualized treatment of NSCLC, especially small molecular targeted therapies that require rapid and accurate molecular genotyping. EGFR-TKIs remain the most important targeted drugs for NSCLC patients with *EGFR*-sensitive mutations and have been recommended, for a number of years, as the standard first-line treatment for these patients by the National Comprehensive Cancer Network [4]. Therefore, to ascertain *EGFR* mutation status after pathological diagnosis is crucial. While, in a real clinical setting, it may not be possible to obtain a tumor specimen from certain patients for pathological examination or it may not be possible to obtain adequate tissue for further molecular analyses, it may also be difficult and unrealistic for patients with NSCLC to undergo re-biopsy after multi-line therapy. Therefore, as a non-invasive test, liquid biopsy has the potential to complement tissue biopsies and is fairly convenient to monitor responses to EGFR-TKI treatment and drug resistance [19, 20]. Circulating tumor DNA (ctDNA) has been approved by the Committee for Medicinal Products for Human Use of the European Medicines Agency [21] to assess *EGFR* mutation status in patients with NSCLC for whom it is not possible to obtain a tumor sample. Indeed, ctDNA exhibits high accuracy for *EGFR* mutation status analysis and is commercially available. Meanwhile, ctDNA analysis has been applied in clinical practice. The sensitivity of ctDNA has been reported to range from 70.0–75.0%, which may be promoted by highly sensitive technologies that are relatively costly for the patients (e.g., digital droplet PCR and next generation sequencing) [21]. Therefore, it is imperative to explore non-invasive, convenient, and economical tumor markers as supplements to predict *EGFR* mutation status and to monitor EGFR-TKI treatment in NSCLC patients. Given their stability in plasma and serum, microRNAs are considered a novel class of non-invasive biomarkers for various types of malignancies [22]. In the present study, we comprehensively evaluated the potential of 3 circulating plasma microRNAs (miR-107, miR-122, and miR-195) as potential novel markers of *EGFR* mutation status in NSCLC patients.

No related information was available as to the two major specific *EGFR* mutation genotypes (*EGFR* exon 19 deletions or *EGFR* p.L858R mutations) that are independently associated with circulating microRNAs. As observed in the clinic and in a study comparing icotinib with gefitinib in previously treated patients with advanced NSCLC [23], the *EGFR* exon 19 deletion was associated with a better response to treatment compared to the *EGFR* p.L858R mutation. To identify potential markers that could distinguish between *EGFR* mutation-positive and *EGFR* wild-type patients and between patients with different *EGFR*-sensitive mutations (*EGFR* exon 19 deletions and *EGFR* p.L858R mutations) would be worthwhile in basic and clinical research. Indeed, in the present study, 3 circulating plasma microRNAs (miR-107, miR-122, and miR-195) were significantly dysregulated in *EGFR* mutation-positive patients compared to *EGFR* wild-type patients.

Initially, microRNA expression profiles were analyzed in the *EGFR* mutation-positive, *EGFR* wild-type, and healthy control cohorts. Seventy-six dysregulated microRNAs were detected in NSCLC patients compared to healthy controls. Further analysis of the 76 dysregulated microRNAs was performed according to the different *EGFR* mutation genotypes. Twenty-one and 5 dysregulated microRNAs were detected in *EGFR* exon 19 deletion and *EGFR* p.L858R mutation patients, respectively, compared to *EGFR* wild-type patients. All 5 dysregulated microRNAs in *EGFR* p.L858R mutation patients were newly discordant microRNAs with large serial numbers (Table 4). Consequently, this cohort of patients was not investigated further. Our study primarily focused on *EGFR* exon 19 deletion patients. This group of patients benefited most from EGFR-TKI treatment. According to the microarray data and related published literature [15–17], 10 microRNAs were screened out for further investigation. Finally, 3 microRNAs (miR-107, miR-122, and miR-195) were identified as being significantly dysregulated.

MiR-107 was significantly upregulated (fold-change: 6.64, *p* < 0.05) in *EGFR* mutation-positive patients compared to *EGFR* wild-type patients. With respect to specific *EGFR* mutation genotypes, miR-107 was upregulated in *EGFR* exon 19 deletion and *EGFR* p.L858R mutation patients compared to *EGFR* wild-type patients (fold-change: 7.44 and 5.22, respectively). However, no significant difference was observed between *EGFR* exon 19 deletion and *EGFR* p.L858R mutation patients (*p* > 0.05). Meanwhile, ROC curve analysis revealed that miR-107 had promising diagnostic potential for distinguishing between *EGFR* mutation-positive and *EGFR* wild-type patients. There are a limited number of studies indicating that miR-107 is dysregulated in NSCLC tissues and cell lines. However, our study is the first to identify miR-107 as a potential marker for *EGFR* mutation-positive patients. As demonstrated in previous studies [24, 25], miR-107 expression is reduced in NSCLC tissues compared to paired normal tissues. MiR-107 functions as a tumor suppressor in NSCLC by targeting brain-derived neurotrophic factor and indirectly regulating the PI3K/AKT signaling pathway. EGFR activation elicits its effects via several downstream signaling pathways, including the PI3K/AKT/mTOR pathway [26]. Thus, we speculate that miR-107 is closely associated with the EGFR pathway and could be a new specific marker for *EGFR*-sensitive mutations. In addition, this could be indirectly validated by its dynamic changes during EGFR-TKI treatment.

MiR-122 and miR-195 have been reported [27, 28] to be dysregulated in *EGFR* mutation-positive patients compared to *EGFR* wild-type patients. However, no study has investigated the differential expression of these 2 circulating microRNAs between specific *EGFR* mutation genotypes. In the present study, miR-122 was significantly upregulated in *EGFR* p.L858R mutation patients compared to *EGFR* wild-type patients. However, no significant difference was detected between *EGFR* exon 19 deletion and *EGFR* wild-type patients. ROC curve analysis demonstrated that miR-122 may serve as a potential specific marker for *EGFR* p.L858R mutation patients (AUC: 0.75). In addition, miR-195, which was significantly downregulated in *EGFR* exon 19 deletion patients compared to *EGFR* wild-type patients, may serve as a potential marker of NSCLC in *EGFR* exon 19 deletion patients (AUC: 0.75). These findings are in agreement with a number of studies focusing on the association of circulating microRNAs with *EGFR*-sensitive mutations. Qiang *et al*. [27] revealed that miR-122 and miR-195 could distinguish between *EGFR* mutation-positive and *EGFR* wild-type patients, and miR-195 was associated with poor differentiation. Another study [28] suggested that circulating miR-122 and miR-195 may have prognostic significance in predicting *EGFR* mutation status and overall survival in female, non-smoking lung adenocarcinoma patients. However, these two studies [27, 28] did not analyze the association between circulating microRNAs and specific *EGFR* mutation genotypes.

Furthermore, we analyzed the diagnostic potential of a combination of microRNAs as a biomarker panel. A combination of miR-107 and miR-195 for discriminating *EGFR* exon 19 deletion patients from *EGFR* wild-type patients produced similar AUC values and higher sensitivity and specificity values than miR-107 or miR-195 alone in the ROC curve analysis. Meanwhile, a panel of miR-107 and miR-122 also produced higher AUC and specificity values for discriminating *EGFR* p.L858R mutation patients from *EGFR* wild-type patients than miR-107 or miR-122 alone; although sensitivity values were lower than miR-122 alone. Taken together, these findings suggest that it may be better to have a panel of 2 microRNAs as markers to distinguish *EGFR* mutation-positive patients from *EGFR* wild-type patients. In addition, multivariate analysis revealed that miR-107 and smoking status are important diagnostic predictors for *EGFR*-sensitive mutations and NSCLC patients with high miR-107 expression levels and no prior history of smoking are more likely to have *EGFR* exon 19 deletion or *EGFR* p.L858R mutations. It has been proven that the distinct profiles of oncogenic mutations, especially *EGFR*, are different between smoking and non-smoking NSCLC patients and this is consistent with the findings of the present study. We are the first to report on miR-107 as a potential marker for *EGFR*-sensitive mutations (*EGFR* exon 19 deletions and *EGFR* p.L858R mutations). To our knowledge, no study has focused on the dynamic changes of circulating plasma microRNAs during the course of EGFR-TKI treatment. Our present findings revealed that the extent of ΔCt variation for miR-107 and miR-195 was significantly correlated with the response to EGFR-TKI treatment, while no significant differences were detected between patients with different PFS times on EGFR-TKI treatment. From the limited number of patients with plasma collected at the time of PD during EGFR-TKI treatment, we found that irrespective of the response of the tumor (CR, PR, SD, or PD), miR-107 and miR-195 expression levels at the time of PD exhibited a tendency to return to pre-treatment levels in the majority of patients. This suggests that circulating plasma microRNAs may have the potential to serve as markers for monitoring EGFR-TKI treatment. In this study, we have only preliminarily observed the objective phenomenon of dynamic changes of microRNAs during EGFR-TKI treatment in a small number of patients from a single institution. Therefore, it is warranted to have well designed large scale studies of patients and to design experiments on tumor cells to determine the precise mechanism(s) of the relationship between microRNA change and EGFR expression in plasma after EGFR-TKI treatment.

This study is limited by the fact that it was performed at a single institution with specific NSCLC patients (e.g., with relatively advanced clinical stages and without a sufficiently large scale of enrolled samples). Further studies are warranted with larger sample sizes and different characteristics to validate the diagnostic potential of circulating plasma microRNAs (miR-107, miR-122, and miR-195) for *EGFR*-sensitive mutations. In addition, cytological experiments on EGFR signaling pathways are required to elucidate the specific functions of these circulating plasma microRNAs. Beyond that, similar studies that have been reported to date that have presented conflicting findings. This may be correlated with ethnical diversity, relatively small sample sizes, and different study technologies. Therefore, it is essential to conduct multicentral studies with standardized methodological protocols to achieve more reliable results.

In conclusion, the present study revealed that upregulation of circulating miR-107 may serve as a potential marker for *EGFR*-sensitive mutations, including *EGFR* exon 19 deletions and *EGFR* p.L858R mutations. In addition, miR-122 may serve as an *EGFR* p.L858R mutation specific marker and miR-195 as an *EGFR* exon 19 deletion specific marker for distinguishing between these patients and *EGFR* wild-type patients. ROC curve analysis suggested that a combined panel of miR-107 and miR-195 or miR-107 and miR-122 had the potential to be a diagnostic marker for *EGFR* exon 19 deletion or *EGFR* p.L858R mutation patients, respectively. Dynamic changes in these 3 microRNAs were also detected during EGFR-TKI treatment, revealing that circulating plasma microRNAs may serve as potential markers for monitoring EGFR-TKI treatment. Our findings highlight the prospective application of circulating plasma microRNAs as non-invasive, convenient, and economical markers for *EGFR*-sensitive mutations.

## MATERIALS AND METHODS

### Patient enrollment

This study was approved by the Ethics Committee of the Affiliated Hospital of the Academy of Military Medical Science, Beijing, China (No. 2012-11-171). NSCLC patients (*n* = 153) were recruited from the Department of Lung Cancer and age and sex-matched healthy controls (*n* = 41) from the Physical Examination Center between December 2014 and April 2016. All study participants have provided informed written consent. Research was conducted in accordance with the 1964 Declaration of Helsinki and its later amendments. The enrolled NSCLC patients were all newly diagnosed with no previous tumor-related therapy and the pathological diagnosis was confirmed by two independent pathologists. Clinical staging was also assessed by two independent professional oncologists according to the American Joint Committee on Cancer/Union for International Cancer Control tumor-node-metastasis staging system (seventh edition) [29]. The performance status of the NSCLC patients was 0–2 according to the criteria of the Eastern Cooperative Oncology Group [30]. All NSCLC patients harboring *EGFR*-sensitive mutations (*EGFR* exon 19 deletions or p.L858R mutations) in tumor tissues received first or second-line EGFR-TKI treatment (gefitinib [250.0 mg], erlotinib [150.0 mg], or icotinib [125.0 mg] daily). Tumor responses were assessed by two independent professional oncologists in accordance with the latest Response Evaluation Criteria in Solid Tumors (version 1.1) [18].

### Plasma collection

For all participants enrolled in the study, 7.0 mL fasted peripheral venous blood samples were collected between 07:00 and 08:00 AM. The plasma was separated within 30 minutes of collection by centrifugation at 4,500 *g* for 10 minutes at 4.0°C and stored immediately at -80.0°C for further analysis.

### Total RNA extraction from plasma samples

Total RNA containing microRNAs was isolated from 200.0 μL of plasma using QIAzol Lysis Reagent (catalogue no. 217184; QIAGEN Inc., Valencia, CA, USA) and purified using the miRNeasy Serum/Plasma Kit (catalogue no. 217184; QIAGEN Inc., Valencia, CA, USA) in accordance with the manufacturer's guidelines.

### Plasma microRNA profiling

Human microRNA Microarrays (Release 21.0; Agilent Technologies Ltd., Santa Clara, CA, USA) containing 2,568 mature microRNA sequences were performed for preliminary screening of dysregulated circulating plasma microRNAs in NSCLC patients with different *EGFR* mutation genotypes. Raw data were extracted using Agilent's Feature Extraction software (version 10.7; Agilent Technologies Ltd., Santa Clara, CA, USA) and normalized using GeneSpring software (Agilent Technologies Ltd., Santa Clara, USA) with the normalization of labeling spike-in RNA and hybridization spike-in RNA according to the manufacturer's protocol.

### qRT-PCR of selected microRNAs

The expression levels of selected microRNAs were analyzed by qRT-PCR using the miScript SYBR Green PCR Kit (catalogue no. 218073; QIAGEN Inc., Valencia, CA, USA). Poly(A)-tailed RNA was used in the qRT-PCR reactions and 5SrRNA was selected as the internal control for data normalization. As previously described [31, 32], a 15.0 μL reaction volume containing total RNA, 0.2 μL of ribonucleotide adenosine triphosphate, 1.5 μL of 10X poly(A) polymerase chain reaction buffer, and 0.2 μL of poly(A) polymerase (catalogue no. M0276L; New England BioLabs Inc., Ipswich, MA, USA) was incubated at 42.0°C for 60 minutes, 70.0°C for 5 minutes, and immediately placed on ice to terminate the reaction. The poly(A)-tailed total RNA was reverse transcribed using 1.0 μg of miR reverse transcription primer (5′-GCG AGC ACA GAA TTA ATA CGA CTC ACT ATA GG(t)18VN-3′), 1.0 μL of ImProm reverse transcriptase (Promega Biotech Co., Ltd., Beijing, China), and reverse transcriptase buffer, according to the manufacturer's protocol. Lastly, qRT-PCR was performed using the miScript SYBR Green PCR Kit (QIAGEN Inc., Valencia, CA, USA). The primer sequences were as follows: 5SrRNA, forward primer: 5′-TCT GAT CTC GGA AGC TAA GCA-3′, reverse primer: 5′-CCT ACA GCA CCC GGT ATT CC-3′; miR-107, forward primer: 5′-AGC AGC ATT GTA CAG GGC TAT CA-3′; miR-122, forward primer: 5′-TGG AGT GTG ACA ATG GTG TTT G-3′; miR-125a-5p, forward primer: 5′-TCC CTG AGA CCC TTT AAC CTG TGA-3′; and miR-195, forward primer: 5′-TAG CAG CAC AGA AAT ATT GGC-3′. All 4 microRNAs shared a common reverse primer (5′-GCG AGC ACA GAA TTA ATA CGA C-3′). QRT-PCR was performed in triplicate in 15.0 μL reaction volumes of PCR master mix using the PCR program on the Stratagene Mx3005P Real-Time PCR Detection System (Agilent Technologies Ltd., Santa Clara, CA, USA). Denaturation was performed at 95.0°C for 10 minutes, followed by 45 cycles of 95.0°C for 15 seconds and 60.0°C for 60 seconds. The raw Ct data were normalized to the expression levels of 5SrRNA using the 2^−Δ(ΔCt)^ method.

### Statistical analyses

The expression of each target microRNA was measured in triplicate and the results were expressed as the mean ΔCt (normalized against 5SrRNA that exhibited relatively stable expression levels across all plasma samples). Subtraction of the Ct value of the target microRNA from 5SrRNA (ΔCt) was calculated. A high ΔCt corresponded to a low microRNA expression level. The ΔCt was then converted to 2^−ΔCt^ to assess alterations in microRNA expression levels between different groups and the fold-change was calculated using the 2^−Δ(ΔCt)^ method. The relative expression of each microRNA was presented as the mean and standard error of the mean. Differences in microRNA expression levels between groups were compared using unpaired Student's *t*-tests or a one-way analysis of variance. Chi-square tests were used for qualitative data. ROC curve analysis was performed to provide an estimate of the target microRNAs’ abilities to discriminate between NSCLC patients with different *EGFR* mutation genotypes. All statistical analyses were conducted using Statistical Package for the Social Sciences for Windows, software version 23.0 (IBM Corp., Armonk, NY, USA). A *p* < 0.05 was considered statistically significant.

## Abbreviations

5SrRNA: 5S ribosomal RNA
AUC: area under the ROC curve
CI: confidence interval
CR: complete response
Ct: quantification cycle
ctDNA: circulating tumor DNA
EGFR-TKI: epidermal growth factor receptor-tyrosine kinase inhibitor
NSCLC: non-small cell lung cancer
PD: progressive disease
PFS: progression-free survival
PR: partial response
qRT-PCR: quantitative real-time reverse-transcription polymerase chain reaction
ROC: receiver operating characteristic
SD: stable disease.
